# The *miR-124* family of microRNAs is crucial for regeneration of the brain and visual system in the planarian *Schmidtea mediterranea*

**DOI:** 10.1242/dev.144758

**Published:** 2017-09-15

**Authors:** Vidyanand Sasidharan, Srujan Marepally, Sarah A. Elliott, Srishti Baid, Vairavan Lakshmanan, Nishtha Nayyar, Dhiru Bansal, Alejandro Sánchez Alvarado, Praveen Kumar Vemula, Dasaradhi Palakodeti

**Affiliations:** 1 Institute for Stem Cell Biology and Regenerative Medicine, GKVK campus, Bangalore, Karnataka 560065, India; 2 Manipal University, Manipal, Karnataka 576104, India; 3 Stowers Institute for Medical Research and Howard Hughes Medical Institute, Kansas City, MO 64110, USA; 4 Department of Neurobiology and Anatomy, University of Utah, Salt Lake City, UT 84112, USA

**Keywords:** Planarian, *Schmidtea mediterranea*, *miR-124*, Brain regeneration, Photoreceptors, Notch, miRNA

## Abstract

Brain regeneration in planarians is mediated by precise spatiotemporal control of gene expression and is crucial for multiple aspects of neurogenesis. However, the mechanisms underpinning the gene regulation essential for brain regeneration are largely unknown. Here, we investigated the role of the *miR-124* family of microRNAs in planarian brain regeneration. The *miR-124* family (*miR-124*) is highly conserved in animals and regulates neurogenesis by facilitating neural differentiation, yet its role in neural wiring and brain organization is not known. We developed a novel method for delivering anti-miRs using liposomes for the functional knockdown of microRNAs. *Smed-miR-124* knockdown revealed a key role for these microRNAs in neuronal organization during planarian brain regeneration. Our results also demonstrated an essential role for *miR-124* in the generation of eye progenitors. Additionally, *miR-124* regulates *Smed-slit-1*, which encodes an axon guidance protein, either by targeting *slit-1* mRNA or, potentially, by modulating the canonical Notch pathway. Together, our results reveal a role for *miR-124* in regulating the regeneration of a functional brain and visual system.

## Abstract (precis)

**Summary:** *miR-124* is required during *de novo* regeneration of the cephalic ganglion and visual system in planarians, as well as in *slit-1* expression in the midline of anterior regenerating tissue via canonical Notch signaling.

## INTRODUCTION

Brain wiring is a complex process that ensures the formation of an intricate network of intercommunicating neurons. This process involves the precise guidance of axons from the neuron to its targets, which, in most animals, occurs during prenatal and early postnatal development (Tessier-Lavigne and Goodman, 1996; Stiles and Jernigan, 2010). Because of their unique capacity to regenerate a complex nervous system, planarian flatworms provide a suitable context in which to study factors essential for brain organization and neural patterning in adult animals. Planarians have a well-organized nervous system, which consists of a bi-lobed brain (cephalic ganglia) and a pair of ventral nerve cords interconnected by commissural neurons (Umesono et al., 1999; Cebria et al., 2002a,b). The eyes are located dorsal to the cephalic ganglia. The planarian eye consists of pigment cells, which form a cup-shaped organ, and photosensing neurons that project rhabdomeres inside the pigment cup, while their axons form the optic chiasm (Sakai et al., 2000; Yamamoto and Agata, 2011). During planarian regeneration, the brain rudiment and eyes form within 24-48 h post-amputation (hpa). Although the differentiating primordia of the brain and eyes are evident ∼3 days post-amputation (dpa), the recovery of the functional brain is completed only after 7 dpa (Inoue et al., 2004; Ong et al., 2016). Axon guidance proteins, such as those encoded by members of the *dscam*, *slit*, *netrin* and *robo* families, are essential for neural regeneration in planarians, and these factors are required for the patterning of the brain (Agata and Umesono, 2008; Yamamoto and Agata, 2011; Fusaoka et al., 2006; Cebria et al., 2007; Beane et al., 2012). However, the factors that regulate the spatiotemporal expression of these genes, which are crucial for the proper patterning of the planarian visual system and brain, are not known.

MicroRNAs (miRNAs) are small RNA species 21-22 nucleotides in length. They function in multiple biological processes, including neural stem cell proliferation, differentiation and patterning (Almuedo-Castillo et al., 2011; Plasterk, 2006; Hatfield et al., 2005; Thatcher and Patton, 2010). Deep sequencing of *Schmidtea mediterranea* small RNAs from the regenerating tissue (blastema) identified the *Smed-miR-124* family as the most prominent miRNAs enriched in the anterior blastema by 3 dpa (Sasidharan et al., 2013). The *miR-124* family is highly conserved across metazoa and is generally enriched in the nervous system (Kosik, 2009). Although the *miR-124* family is highly expressed in the adult brain of vertebrates, its role in adult neurogenesis is not known. Since whole-mount *in situ* hybridization (WISH) revealed that the *miR-124* family is enriched in the nervous system of the adult planarian (Gonzalez-Estevez et al., 2009; Sasidharan et al., 2013), we aimed to explore the function of this family, including its potential roles in neurogenesis.

We developed a novel liposome-based method for efficient delivery of *anti-miR-124* into live planarians to study the function of the *miR-124* family (hereafter collectively referred to as *miR-124*). Extensive characterization of *miR-124* knockdown (KD) phenotypes, which include severe brain and eye defects, revealed an essential role for *miR-124* in the generation and/or maintenance of discrete neural populations and eye progenitors, in addition to the patterning of neurons in the anteriorly regenerating planarian. Furthermore, *miR-124* KD caused an expansion of *Smed-slit-1* expression in the midline.

Target prediction analyses identified a cohort of genes essential for axon guidance, planar cell polarity, and the Notch pathway as likely targets of *miR-124*. To preliminarily validate our target predictions and gain insights into the mechanism of action of *miR-124*, we focused our efforts on the Notch pathway. The KD of Notch pathway genes recapitulated the midline defects observed after *miR-124* KD, suggesting that *miR-124* might regulate *slit-1* expression via the canonical Notch pathway. Altogether, our study uncovered roles for *miR-124* in neurogenesis and neuronal organization during adult brain and eye regeneration. Furthermore, we find that *miR-124* is likely to be required for midline patterning through a deeply evolutionarily conserved *miR-124/*Notch regulatory interaction.

## RESULTS

### Expression of the planarian *miR-124* family is enriched in the cephalic ganglia and ventral nerve cords

**Fig. 1.**
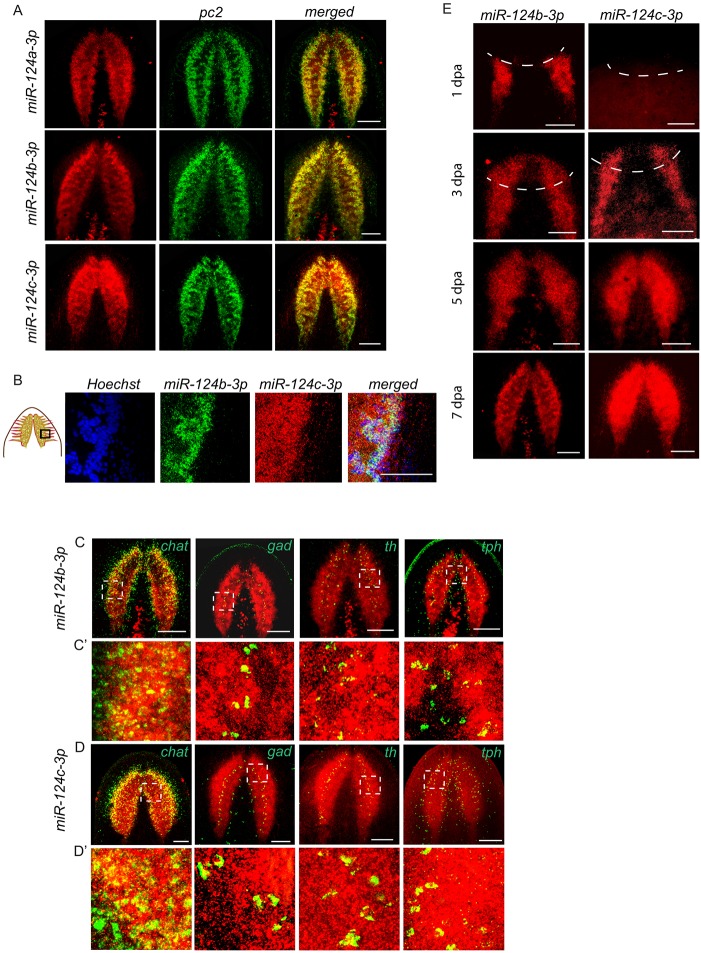
**Expression of *miR-124* family miRNAs in the planarian CNS.** (A) Double fluorescent *in situ* hybridization (FISH) colocalization for *miR-124* family miRNAs with the pan-neuronal marker *pc2*. The merged images of double FISH show the expression of the miRNAs in the cephalic ganglia. (B) Differential expression of *miR-124* family miRNAs in the region indicated. (C-D′) *miR-124b* and *miR-124c* colocalize with different neuronal subtypes. (C,D) Double FISH was performed for *miR-124b* or *miR-124c* and cholinergic (*chat*), GABAergic (*gad*), dopaminergic (*th*) and serotonergic (*tph*) neuronal markers. (C′,D′) Higher magnification of the boxed regions from C and D. (E) *miR-124b* and *miR-124c* expression during regeneration. Dashed lines indicate the site of amputation. Scale bars: 100 µm.

The planarian genome encodes five *miR-124* family members (*Smed-miR-124a*, *b*, *c*, *d* and *e*), which differ from each other after the tenth nucleotide from the 5′ end (Friedlander et al., 2009; Lu et al., 2009; Palakodeti et al., 2006). High-throughput sequencing of small RNAs from the anterior blastema of regenerating animals detected enrichment for *miR-124a*, *b*, *c* and *e* (Sasidharan et al., 2013). WISH using locked nucleic acid (LNA) detection probes confirmed the expression of *miR-124a*, *b* and *c* in the cephalic ganglia and ventral nerve cords (Fig. S1A). The *miR-124e* detection probe was not used for further expression studies because of its weak and inconsistent staining. Colocalization of *miR-124a*, *b* and *c* with the pan-neural marker *Smed*-*pc2* and Hoechst staining revealed their expression in the bi-lobed cephalic ganglia. Unlike *miR-124a* and *b*, *miR-124c* showed a distinct expression pattern in the brain and ventral nerve cord, suggesting that the LNA probes effectively differentiate the expression of *miR-124c* from that of *miR-124a* and *b* (Fig. 1A,B, Fig. S1A-C). Additionally, *miR-124c* showed broad expression in both the perinuclear soma and the neuronal axon bundles in the bi-lobed cephalic ganglia, as compared with *miR-124a* and *b*, which were enriched in the perinuclear soma (Fig. 1B, Fig. S1B,C). Since *miR-124a* and *b* showed similar expression patterns, all additional expression studies were performed using the *miR-124b* probe. Colocalization studies of *miR-124b* and *miR-124c* with other neuronal markers, including *choline acetyltransferase* (*Smed-chat*), *glutamate decarboxylase* (*Smed-gad*), *tyrosine hydroxylase* (*Smed-th*) and *tryptophan hydroxylase* (*Smed-tph*), revealed broad expression of *miR-124* in all of these neural subtypes (Fig. 1C,D, Fig. S2A,B).

Further examination of *miR-124b* and *miR-124c* expression at different regeneration time points (1, 3, 5 and 7 dpa) revealed their enrichment in the brain primordia as early as 3 dpa (Fig. 1E). Enrichment of *miR-124b* and *miR-124c* was also observed in the regenerating brain at 5 and 7 dpa, as well as in the anterior commissure that connects the bi-lobed structure (Fig. 1E). The expression of *miR-124c* in the pre-existing ventral nerve cords close to the regenerating brain was not seen until 3 dpa, whereas the expression of *miR-124b* was evident by 1 dpa (Fig. 1E). However, weak *miR-124c* expression was seen throughout the pre-existing ventral nerve cord between 3 and 7 dpa. Prominent expression of *miR-124c* throughout the ventral nerve cords was observed only after 7 dpa (Fig. S1D). Interestingly, the brain and eye primordia are known to begin forming between 1 and 2 dpa. Since none of the *miR-124* genes showed enrichment in the blastema until 3 dpa, this suggests that these miRNAs might contribute to the production of neural lineages born after 3 dpa and/or play roles in later stages of neural regeneration. To test these possibilities, we aimed to perturb the function of *miR-124* during planarian regeneration.

### Design of non-viral liposomes for enhanced delivery of anti-miRs in planarians

Currently, the study of miRNAs in planarians is limited by the lack of effective methods for delivering anti-miRs into cells. Several methods, such as microinjection and soaking, have been used to deliver double-stranded RNA (dsRNA) into planarians (Orii et al., 2003; Sánchez Alvarado and Newmark, 1999; Martin-Duran et al., 2008; Gonzalez-Estevez et al., 2003). However, these methods have not proved to be successful for delivering anti-miRs (data not shown). To overcome this, we custom-designed cationic, non-viral liposomal vectors to deliver anti-miRs into planarian cells *in vivo*.

**Fig. 2.**
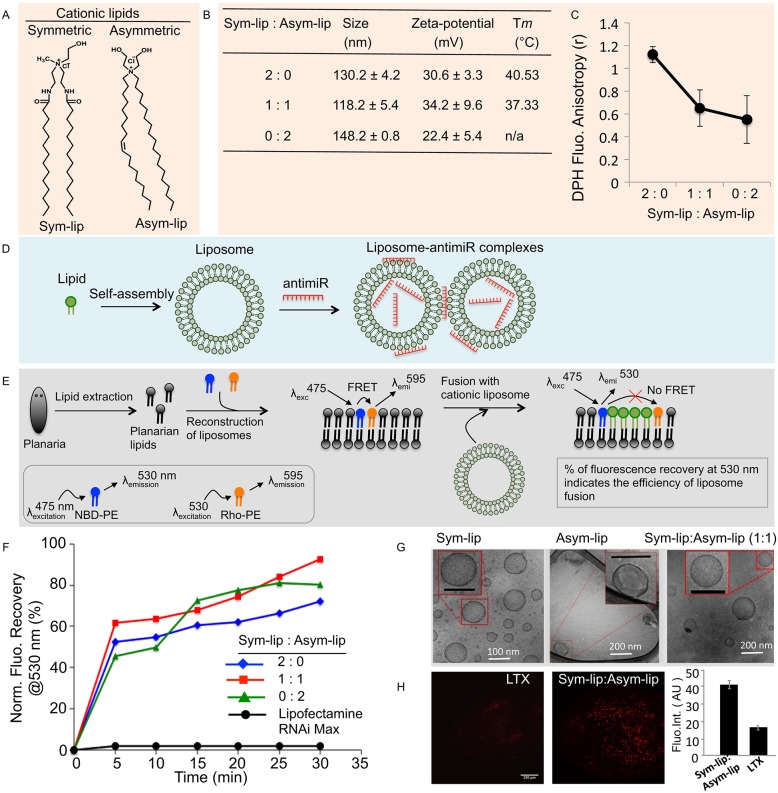
**Biophysical characterization of liposomes/lipoplexes and their fusogenicity with membranes.** (A) Structures of symmetric and asymmetric cationic lipids. (B) Biophysical parameters of liposomes prepared using various ratios of lipids. (C) Fluorescence anisotropy measurement of diphenylhexatriene (DPH) in three types of liposomes, showing higher membrane fluidity of liposomes prepared using either asymmetric lipid or a 1:1 ratio of Sym-Lip and Asym-Lip. Error bars indicate s.d. (D) Schematic of lipoplex formation with anti-miRs and liposomes. (E) Scheme for reconstitution of the membrane using planarian lipids and fluorophore-tagged lipids (FRET pair of NBD-PE and Rho-PE). The proximity of the two fluorescent lipids in reconstituted planarian membrane enables FRET. Fusion of liposomes leads to separation of the fluorophores and inhibits FRET. Thus, emission of NBD-PE can be recovered. (F) Recovery of emission fluorescence at 530 nm indicates the higher fusogenic ability of cationic lipids over Lipofectamine. (G) TEM images of liposomes. (H) Confocal microscopy of planarians that were transfected with fluorescent dye-tagged anti-miRs using either the commercial agent Lipofectamine LTX or cationic liposomes. The bar chart shows the fluorescence intensity of Lipofectamine LTX and cationic liposomes (calculated using ImageJ). Error bars indicate s.d. Scale bar: 200 μm.

We designed cationic liposomes that are fluidic at lower temperatures (∼25°C) to efficiently fuse with planarian cells. Cationic lipids with symmetric (di-octadecyl, Sym-Lip) and asymmetric (octadecyl and oleyl, Asym-Lip) hydrophobic chains were synthesized and characterized by mass spectrometry (Fig. 2A, Fig. S3A-F). The fluidity of liposomes was modulated by their preparation using a single lipid [either Sym-Lip (symmetric liposomes) or Asym-Lip (asymmetric liposomes)] or a 1:1 molar ratio of symmetric-asymmetric lipids (co-liposomes), along with cholesterol. The biophysical characterization of these three liposomes, including their size, surface charge and fluidity, revealed that co-liposomes are the best carriers of nucleic acids compared with symmetric and asymmetric liposomes (Fig. 2B,C). Furthermore, gel retardation studies of the three types of liposomes mixed with miRNAs at different concentrations revealed that all three liposomes efficiently complex with miRNA at a higher lipid-to-miRNA charge ratio of 20:1, inhibiting liposome mobility in the gel (Fig. 2D, Fig. S4A).

To evaluate the fusogenicity of cationic liposomes, we performed fluorescence resonance energy transfer (FRET) by reconstituting the liposome with planarian lipids (see Materials and Methods) using dual fluorophore lipids (NBD-PE and Rho-PE) (Fig. 2E). NBD-PE and Rho-PE in proximity have an emission wavelength of 595 nm due to FRET (Mukthavaram et al., 2009). Fusion of the liposome with the membrane leads to a spatial separation between NBD-PE and Rho-PE lipids, which results in emission at 530 nm instead of 595 nm owing to the absence of FRET. The total percentage of fluorescence recovery at 530 nm, as a direct measure of the fusogenicity of the liposomes (Fig. 2E,F), was 72%, 80% and 93% for the symmetric, asymmetric and co-liposomes, respectively. Additionally, no significant recovery (<5%) was observed for Lipofectamine RNAi Max (Thermo) (Fig. 2F). These data suggest that the cationic liposomes and co-liposomes were particularly efficient at inducing fusion with planarian lipid membranes when compared with Lipofectamine RNAi Max.

Transmission electron microscopy (TEM) was performed on the three liposomal formulations (Fig. 2G) and lipoplexes containing oligonucleotides*.* This demonstrated the unilamellar stable nature of the liposomes (Fig. S4B). Furthermore, fluorescently labeled oligonucleotide complexes with co-liposomes and Lipofectamine LTX were incubated with regenerating planarians, and confocal imaging revealed that co-liposomes have a better penetrance into tissues than Lipofectamine LTX (Thermo) (Fig. 2H, Fig. S5A). Confocal imaging of cell macerates from animals treated with co-liposomes further demonstrated that fluorescently labeled oligonucleotides successfully penetrate into the cytoplasm and nuclei of the cells (Fig. S5B). Cumulatively, these data indicate that these custom-designed liposomes could be highly efficient delivery vehicles for nucleic acids in planarians.

### Knockdown of *ovo* and multiple miRNAs using non-viral liposomes

**Fig. 3.**
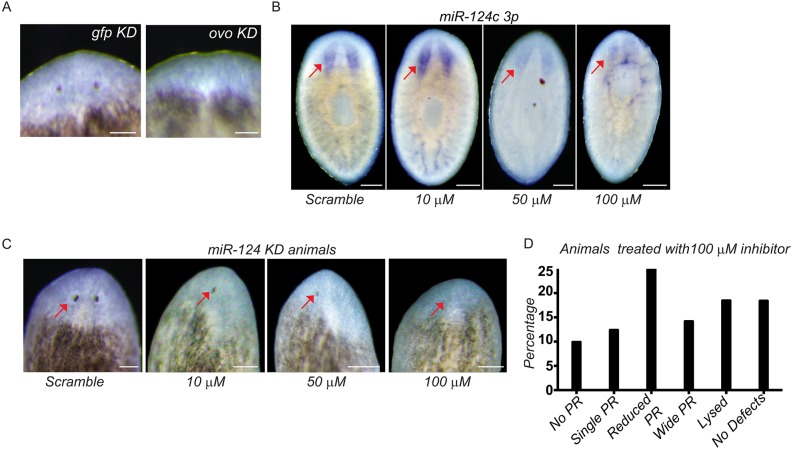
**Validation of liposome-mediated *ovo* and *miR-124* KD.** (A) *ovo(RNAi)* animals (15/15) showed an eyeless regeneration phenotype in the first round of anterior regeneration after 7 dpa. (B) WISH showing the expression of *miR-124c* at 7 dpa during the second round of regeneration in KD animals (*n*=5). (C) Animals treated with 100 μM scrambled miRs rarely showed regeneration defects (4/90, 4.4%), whereas 10 μM (7/33, 21.2%), 50 μM (12/33, 36%) or 100 μM (91/112, 81%) anti-miR-treated animals frequently showed eye regeneration defects. Arrows indicate eye in the scrambled-treated or anti-miR-treated animals. Scale bars: 100 µm. (D) The percentage of animals that show various categories of photoreceptor (PR) defects observed in regenerated animals following 100 µM anti-miR treatment.

To validate the effectiveness of our liposome-based nucleic acid delivery method, we performed *ovo* KD. *ovo(RNAi)* animals are known to lack eyes. As expected, delivery of *ovo* dsRNA using liposomes resulted in an eyeless phenotype in 100% of the treated animals (*n*=15), whereas *gfp*-treated controls regenerated eyes normally (*n*=15) (Fig. 3A). This is on a par with the phenotype severity and penetrance obtained with dsRNA feeding or injection delivery methods (Lapan and Reddien, 2012).

Next, we examined the effectiveness of our delivery method for knocking down miRNAs. We administered an anti-miR that was predicted to target all *miR-124* family members using co-liposomes in regenerating animals at 1 dpa. Animals were treated with anti-miRs at 10, 50 and 100 µM, and a scrambled anti-miR negative control was used at 100 µM.

The miRNA KD was verified by examining expression of one of the *miR-124* family members, *miR-124c*, by WISH using antisense LNA probes. The animals treated with 50 or 100 µM anti-miRs showed significant decreases in expression of *miR-124c* (Fig. 3B). Although we observed a reduction in the expression of *miR-124c* in the 50 µM treatment condition, there was no obvious disorganization of the brain or visual system in these animals (Fig. S5C). This suggests that the reduction in *miR-124c* levels is not the result of a loss of brain tissue, but is specific to the inhibition of expression by the anti-miRs.

Since the anti-miRs are predicted to target all *miR-124* family members, we also examined the expression levels of another member of the family, *miR-124b.* As expected, we observed a significant decrease in *miR-124b* expression in the animals treated with 100 μM anti-miRs (Fig. S6A), suggesting that all *miR-124* members are likely to be inhibited. To assess whether the decreased levels of *miR-124* were due to reduction in brain size, we performed WISH for *miR-124a* and *miR-124c* on both control and KD animals after the first round of regeneration, when there were no observable defects in brain morphology (Fig. S6B). We detected a significant reduction in the levels of the two miRNAs, even though the cephalic ganglia were clearly present, suggesting that the reduction in miRNA levels is not the result of a smaller brain.

To confirm this, we performed qPCR for each *miR-124* family member after treatment with either the scrambled or anti-miRs. The anti-miR-treated animals showed a 50-90%, 30-40% and 20-40% decrease in the levels of *miR-124a*, *b* and *c*, respectively (Fig. S6C). This suggested that our liposome-based delivery method is robust.

### Liposome-mediated miRNA KD generates highly penetrant and specific regeneration phenotypes

To determine whether the observed KD of miRNA expression translated into robust and specific regeneration defects in planarians, we next examined the live phenotypes of planarians treated with *anti-miR-124.* Encouragingly, 81% of the animals treated with 100 µM anti-miRs showed various types of eye defects, including small eyes (25%), the complete absence of eyes (10%), cyclopia (13%), and eyes spaced abnormally far apart (14%) (Fig. 3C,D, Fig. S6D). A few animals (19%) also showed lesions in the head region and subsequently lysed (Fig. 3D).

To rule out the possibility that these regeneration defects might be due to unanticipated non-specific effects, we also treated planarians with anti-miRs targeting the *miR-10* family. We observed a phenotype entirely different from that of *miR-124* KD animals. No defects in the regeneration of the eyes or brain were observed. Instead, the regeneration of the intestine was disrupted, resulting in fusion of the two posterior gut branches (Fig. S6E,F).

Cumulatively, these validation experiments indicate that our liposome-based technique is highly specific and robust for delivering anti-miRs into planarian cells for functional studies.

### *miR-124* is essential for regeneration of the eye in planarians

**Fig. 4.**
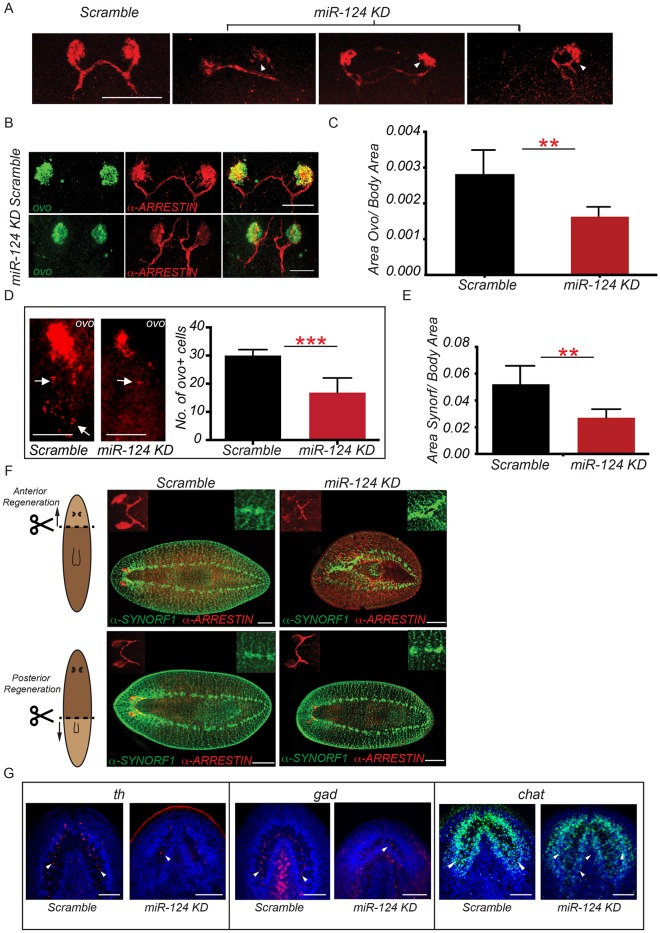
***miR-124* KD disrupts regeneration of the eyes and brain.** (A) Immunostaining of the optic chiasm (anti-ARRESTIN) in scrambled-treated versus *anti-miR-124*-treated animals (100 μM each) at 7 dpa after the second round of regeneration. 13/16 KD animals showed diverse eye defects. Arrowheads indicate defects in KD animals. (B) FISH showing the expression of *ovo* in the eye and immunostaining for photosensory neurons (anti-ARRESTIN) in *miR-124* KD (*n*=10) and scrambled-treated (*n*=7) animals. (C) Quantification of the size of the eyes determined by the ratio of the area of *ovo* staining to the total body area in scrambled*-*treated (*n*=6) versus *anti-miR-124-*treated (*n*=5) animals. (D) FISH showing the expression of *ovo* in the eye and trail cells posterior to the eye in scrambled-treated and *miR-124* KD animals at 4 dpa (*n*=6 animals per treatment condition). *ovo^+^* trail cells were quantified in each condition. (E) Quantification of anti-SYNORF-1 staining of anteriorly regenerating animals normalized to body area in scrambled-treated versus *anti-miR-124*-treated (100 μM) animals (*n*=5 each). (F) Organization of the brain, ventral nerve cords, (anti-SYNORF-1, green) and photosensory neurons (anti-ARRESTIN, red) in scrambled (10/10) versus *miR-124* KD animals regenerating either a head (10/10; top) or tail (13/16; bottom). Illustrations show the amputation plane (dashed line) and lighter colored areas indicate regenerated tissue. (G) FISH for neuronal subtypes in the brain of scrambled-treated versus *miR-124* KD animals, including dopaminergic (*th*, red; *n*=6), GABAergic (*gad*, red; *n*=6) and cholinergic neurons (*chat*, green; *n*=6). Nuclei are stained with Hoechst (blue). Arrowheads indicate the neural subtypes. **P<0.01, ***P<0.001 (Student's *t*-test). Error bars indicate s.d. Scale bars: 50 µm in A,B,D; 100 µm in F,G.

Since we observed obvious eye defects in live animals, we examined the morphology of the regenerated optic cup in *miR-124* KD animals. Using an anti-ARRESTIN antibody that labels the cell bodies, rhabdomeres and axons of the photoreceptor neurons (PRNs), we observed that *miR-124* KD animals displayed obvious fasciculation and elongation defects of the axons. The optic chiasm appeared highly disorganized (Fig. 4A,B). Furthermore, the aggregation of PRN cell bodies appeared significantly reduced in *miR-124* KD animals compared with scrambled controls (Fig. 4A, arrowheads), suggesting that this differentiated cell population might be missing after loss of *miR-124*.

Since we suspected that the numbers of differentiated cells of the eye were decreased in *miR-124* KD animals, we quantified the size of the regenerated eyes. We performed FISH for *ovo*, which labels the entire eye lineage, including the terminally differentiated cells of the pigment cup and PRNs (Lapan and Reddien, 2012). The size of the eyes was quantitated by measuring the area of *ovo* staining in the differentiated eye normalized to the total body area or brain size (Fig. 4C, Fig. S7A). As expected, *miR-124* KD animals showed a significant decrease in the overall size of the regenerated eyes compared with scrambled-treated controls, suggesting a reduction in the number of differentiated cells. To confirm this, we counted individual ARRESTIN^+^ PRNs in the regenerated eyes. There were significantly fewer ARRESTIN^+^ cells in *miR-124* KD animals than in the scrambled-treated controls, confirming that this cell population was lost (Fig. S7B).

A reduction in the number of differentiated cells could mean that eye progenitors are lost after KD of *miR-124.* Therefore, we quantified the number of eye progenitors at 4 dpa, which are defined as *ovo^+^* ʻtrail' cells posterior to the eye primordia (Lapan and Reddien, 2012). Indeed, the number of *ovo^+^* trail cells was significantly reduced in *miR-124* KD animals (Fig. 4D), suggesting that *miR-124* may be required for the specification, differentiation and/or survival of eye progenitors.

### *miR-124* is required for regeneration of the cephalic ganglia

Since *miR-124* family members are highly enriched in the nervous system, we examined whether brain regeneration was affected by loss of these miRNAs. Indeed, some *miR-124* KD animals (16%) displayed a reduction in the overall size of the brain compared with scrambled-treated controls, which was evident by immunostaining for the broad neural marker SYNORF-1 (Fig. 4E,F).

The planarian brain is composed of different neuronal subtypes (Roberts-Galbraith et al., 2016; Cowles et al., 2013; Ong et al., 2016). To explore the neural defects, we examined three of the major neuronal subtypes in planarians after *miR-124* KD: the dopaminergic (*th*^+^), GABAergic (*gad*^+^) and cholinergic (*chat*^+^) neurons. FISH revealed a dramatic decrease in the numbers of *gad*^+^ and *th*^+^ cells in *miR-124* KD animals compared with scrambled-treated controls, suggesting a loss of GABAergic and dopaminergic neurons. Interestingly, KD of *miR-124* caused a significant increase in the number of *chat*^+^ cells, indicating a likely expansion of cholinergic neurons (Fig. 4G, Fig. S7C).

Since a disruption in differentiated neural cell types and a reduction in the size of the brain might indicate defects in the generation and/or maintenance of neural progenitors, we examined the expression of a progenitor marker for the nervous system (*Smed-pax6A*) (Wenemoser et al., 2012). In *miR-124* KD animals, no reduction in the expression of *pax6A* was observed at 4 dpa (Fig. S7D). However, the KD animals showed mispatterned expression of *pax6A^+^* cells. This suggested that *miR-124* might not be required for the initial generation of neural progenitors, but may instead be required later during the progression of the lineage. Cumulatively, these data indicate that *miR-124* may be involved in the production of discrete neural subpopulations during planarian brain regeneration.

We also tested whether *miR-124* is required in regeneration contexts that do not involve rebuilding organs from scratch. First, we examined the head fragments that only regenerate tissues posterior to the brain *de novo*. In these animals, the pre-existing brain and eyes undergo remodeling and resizing, which is dependent on tissue turnover (Gurley et al., 2010). *miR-124* KD animals regenerating posterior tissue showed no defects in the brain, eyes or optic chiasm, suggesting that *miR-124* is not required for brain remodeling in posteriorly regenerating animals (Fig. 4F).

We also examined these organs during homeostatic tissue turnover after *miR-124* KD. Intact animals were continuously treated with anti-miR for 1 month. No defects in the organization of the nervous system or eyes were observed after *miR-124* KD compared with scrambled-treated controls (Fig. S7E). These results suggest that *miR-124* is primarily required during *de novo* regeneration of the cephalic ganglia, ventral nerve cords and visual system in planarians.

### *miR-124* eye regeneration defects are independent of brain defects

**Fig. 5.**
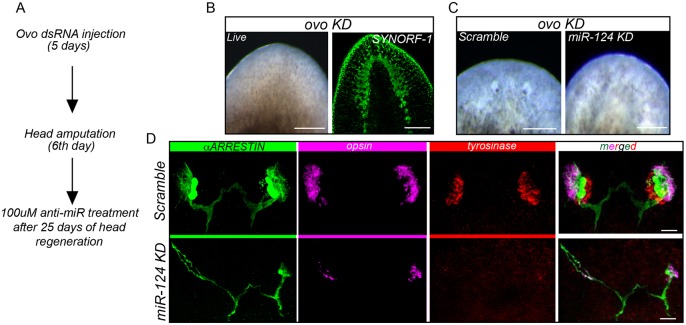
***miR-124* is required for eye-specific regeneration.** (A) Methodology of eye-specific regeneration experiments. *ovo* dsRNA was administered by injection for 5 days, and on the sixth day the animals were amputated pre-pharyngeally. LNA anti-miRs were administered on alternative days for 8 days after 25 days of head regeneration. (B) Live images and anti-SYNORF-1 staining of *ovo* KD animals after 25 days of RNAi (*n*=15 animals). (C) Live images of *ovo* KD animals showing recovery of eyes after scrambled treatment (*n*=15/15 recovery) but not after *anti-miR-124* treatment (*n*=12/15 failure). (D) Molecular characterization of eye phenotypes using anti-ARRESTIN (photosensory neurons and axons), *opsin* (photosensory neuron cell bodies) and *tyrosinase* (pigment cup cells). *miR-124* KD animals (10/12) showed drastic reduction in *opsin* and *tyrosinase* staining, along with mispatterned photoreceptor neurons. Scale bars: 100 µm in B; 50 µm in C,D.

Brain malformations are known to cause secondary defects in the eyes of planarians (Cowles et al., 2013). Therefore, it is possible that the observed eye defects upon *miR-124* KD might be secondary to abnormalities present in the brain. To help address this, we examined the function of *miR-124* during eye-only regeneration in the absence of any brain defects. This was achieved by first generating *ovo(RNAi)* worms, which lacked eyes but did not display brain abnormalities or any other morphological defects (Lapan and Reddien, 2012) (Fig. 5A,B). Next, we stopped the treatment with *ovo* dsRNA, which allowed the eyes to progressively regenerate over the course of 1 month as the gene expression recovered*.* During this recovery period, we treated animals with either scrambled or *anti-miR-124*, and assessed whether the eyes could regenerate in the presence of otherwise normal anatomy (Fig. 5A,B). The scrambled-treated live animals clearly regenerated eyes, whereas the animals treated with *anti-miR-124* showed no signs of eye recovery at the same time point (Fig. 5C). To confirm this, we visualized the differentiated PRNs (*Smed-opsin*), optic chiasm (anti-ARRESTIN) and pigment cells (*Smed*-*tyrosinase*) in fixed worms. Consistent with our live animal observations, scrambled-treated worms regenerated both PRNs and pigment cells, in addition to a well-organized optic chiasm. By contrast, *miR-124* KD animals possessed significantly fewer mature pigment cup cells and PRNs. Additionally, the axons projected by the few PRNs that were present after *miR-124* KD did form an optic chiasm, but mistargeting of some axons was observed (Fig. 5D). Cumulatively, the results of this eye-specific regeneration paradigm suggest that *miR-124* has a direct role in eye regeneration, and this eye phenotype is unlikely to be a secondary effect of a generalized brain defect.

### The putative targets of *miR-124* are crucial for neuronal organization and growth

In an effort to identify the transcripts regulated by *miR-124*, we performed target prediction using the miRanda program (Enright et al., 2003). Target prediction identified a total of 1699 putative targets that might be regulated by the *miR-124* family (Table S1). Although most of the predicted targets were common between all the *miR-124* family miRNAs, we identified 128 and 125 predicted targets unique to *miR-124b* and *miR-124c*, respectively. Gene set enrichment analysis (GSEA) (Subramaniam et al., 2005; Mootha et al., 2003) of *miR-124* targets identified genes normally involved in axon guidance (*slit-1*, *dscam*, *netrin-1*), Notch signaling (*notch-2*, *jagged-2*) and planar cell polarity (PCP) (*dishevelled-2*, *daam-1*) (Table S2, Fig. S8A). Most have been shown to regulate visual neuronal organization and neuronal growth in planarians (Yamamoto and Agata, 2011; Fusaoka et al., 2006; Cebria et al., 2007; Gurley et al., 2008; Almuedo-Castillo et al., 2011; Beane et al., 2012).

**Fig. 6.**
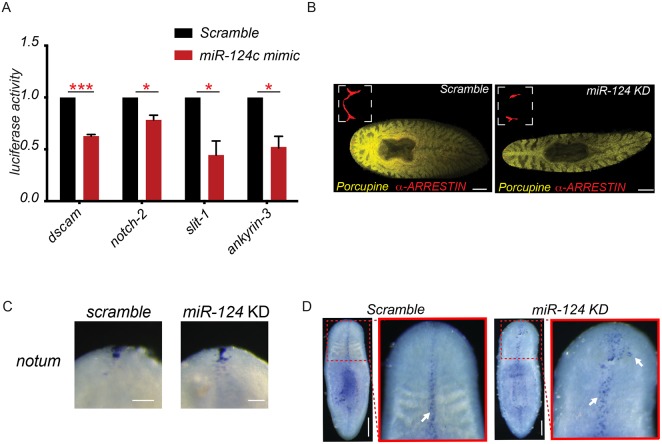
**Target prediction to identify *miR-124* targets.** (A) Luciferase assay was carried out to validate miRNA targets. *P<0.05, ***P<0.001 (Student's *t*-test). Error bars indicate s.d. (B) FISH for *porcupine-1* revealed that the organization of the intestine is indistinguishable between scrambled*-*treated and *anti-miR-124*-treated animals. (C) WISH for *notum* in scrambled-treated and *anti-miR-124*-treated animals (D) Colorimetric WISH showing *slit*-*1* expression in scrambled-treated and anti-*miR-124*-treated animals at 7 dpa from the second round of regeneration. Arrows indicate the expression of *slit-1* in the midline region. Scale bars: 100 µm.

We validated some of the predicted targets of *miR-124*, including *dscam*, *slit-1*, *ankyrin-3* and *notch-2*, by cloning their miRNA binding sites into the 3′-UTR region of the Firefly luciferase gene, followed by transfection into HEK293 cells. Upon *miR-124* mimic transfection, we observed a 24-50% decrease (*notch-2*, 24%; *dscam*, 37%; *slit-1*, 50%; *ankyrin-3*, 43%; *P*<0.05) in Firefly luciferase activity compared with controls (Fig. 6A, Fig. S8B). These results show that the tested 3′-UTRs are likely targets of *miR-124 in vivo*.

Furthermore, we performed transcriptome sequencing to determine the transcriptional changes following *miR-124* KD. The transcriptome sequencing identified 2609 transcripts that were differentially expressed (*P*<0.05). Of these, 1523 were significantly upregulated (Table S3). We used qPCR to examine the expression levels of 30 of these transcripts, and validated 25 that were significantly upregulated after *miR-124* KD (*P*<0.05; Fig. S8C). The other five were upregulated by more than 2-fold, showing a similar trend to the transcriptome data, although not statistically significant. Among the 25 validated transcripts, nine were predicted as targets by miRanda. Interestingly, our data also show that most of the miRanda predicted targets were either marginally upregulated or show no change in expression upon *miR-124* KD. It has previously been reported that *miR-124* could potentially function by repressing mRNA translation rather than promoting transcript degradation (Hendrickson et al., 2009). Here we hypothesize that most of the targets of *miR-124* that are only marginally upregulated upon *miR-124* KD might be regulated by translational repression.

### Mediolateral polarity is disrupted by loss of *miR-124*

The disruption of anteroposterior (A/P) or mediolateral (M/L) polarity might cause the observed eye and brain defects in *miR-124* KD animals (Gurley et al., 2008). To determine whether the *miR-124* KD phenotype is secondary to a polarity defect, we first investigated the organization of an organ system that is often disrupted by perturbation of these axes: the intestine (*Smed-porcn-1^+^*) (Rink et al., 2009; Petersen and Reddien, 2009; Gurley et al., 2010; Cebria et al., 2007). We found that the intestines were indistinguishable between *miR-124* KD animals and controls (Fig. 6B). No fusion of the posterior gut branches, ectopic or missing pharyngeal cavities, or additional major gut branches were observed, which are typical defects in worms with generalized A/P or M/L disruption.

We next examined whether A/P and M/L markers were likely to be targets of *miR-124*. Furthermore, we performed WISH to check for the expression of *Smed-notum*, an anterior marker and key regulator of A/P polarity (Petersen and Reddien, 2011), at 2 dpa in scrambled-treated versus *anti*-*miR-124*-treated animals (Fig. 6C). No difference in *notum* expression was observed between the control and KD animals. Cumulatively, this suggests that A/P polarity is not disrupted by *miR-124* KD.

By contrast, *slit-1*, a broad marker for the midline, was a predicted target of *miR-124*. WISH for *slit-1* revealed the presence of ectopic *slit-1^+^* cells in the anterior blastema after KD of *miR-124* (Fig. 6D). Interestingly, the loss of *slit-1*, not its expansion, is typically associated with a reduction in brain size and cyclopia (Cebria et al., 2007) like that we observe after *miR-124* KD. These data suggest that the eye and nervous system abnormalities in *miR-124* KD animals are unlikely to be secondary to a generalized loss of A/P or M/L polarity.

### *miR-124* may regulate the *slit-1-*expressing midline via canonical Notch signaling

Finally, we aimed to provide functional evidence that this list of predicted *miR-124* targets can yield insights into the undoubtedly complex functions of this miRNA family during planarian regeneration. As a pilot experiment, we selected *notch-2* as a candidate for functional analysis because a previous RNAi screen in the lab indicated that canonical Notch signaling might be involved in regulation of the midline, which we knew to be disrupted after *miR-124* KD.

**Fig. 7.**
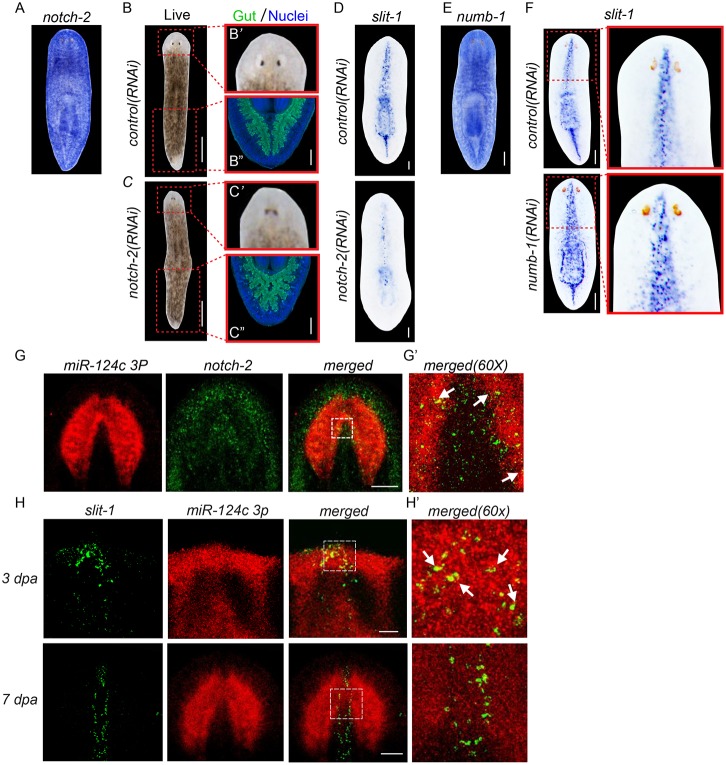
**Regulation of *notch-2* and *slit-1* by *miR-124*.** (A) Colorimetric WISH shows broad expression of *notch-2* in uninjured planarian. (B-C″) *notch-2* KD leads to cyclopia (B′ versus C′; 14 dpa) and fusion of posterior gut branches (B″ versus C″; *mat*, green; DAPI, blue; 17 dpa). (D) Colorimetric WISH for *slit-1* after long-term *notch-2* RNAi. *slit-1* is significantly reduced in *notch-2(RNAi)* planarians compared with controls. (E) Colorimetric WISH shows broad expression of *numb-1* in uninjured planarian. (F) Colorimetric WISH for *slit-1* in control versus *numb-1(RNAi)* planarians at 23 dpa. Higher magnification images of the head show ectopic *slit-1*^+^ cells in *numb-1(RNAi)* planarians. (G,G′) Double FISH showing colocalization (arrows) of *miR-124c* (red) and *notch-2* (green). (H,H′) Double FISH for *slit-1* and *miR-124c* during anterior regeneration. *miR-124c* colocalizes with *slit-1* (arrows) at 3 dpa, but not at 7 dpa. Scale bars: 100 µm.

Knockdown of *notch-2*, which is broadly expressed throughout the planarian body (Fig. 7A), resulted in conspicuous regeneration defects that are typically associated with disruption of the midline. Specifically, *notch-2(RNAi)* animals displayed cyclopia and fusion of the two posterior gut branches (Fig. 7B,C). Since these defects are similar to those reported after RNAi of *slit-1* (Cebria et al., 2007), we examined the expression of *slit-1* after RNAi of *notch-2*. As expected, *notch-2(RNAi)* animals displayed a significant reduction in the number of *slit-1^+^* cells (Fig. 7D). Interestingly, RNAi of other components of the canonical Notch pathway, including the core transcription factor *Su(H)* and the ligand *delta-3*, resulted in very similar midline defects. These included cyclopia, fusion of the posterior gut branches, and loss of *slit-1* expression (Fig. S9A-J). Furthermore, RNAi of a negative regulator of Notch signaling, *numb-1*, yielded the opposite phenotype: a conspicuous increase in *slit-1*^+^ cells (Fig. 7E,F), which appeared strikingly similar to the phenotype observed for *slit-1* expression after *miR-124* KD.

Next, we investigated whether the effects on *slit-1* expression are likely to be direct effects of *notch-2/miR-124* regulation. We first examined colocalization of the *notch-2* receptor and the *delta-3* ligand in the *slit-1^+^* population. This revealed that *notch-2* and *delta-3* are both expressed in discrete subpopulations of *slit-1^+^* cells and in cells directly adjacent to *slit-1^+^* cells (Fig. S10). Since Notch/Delta signaling is cell-autonomous, this suggests the possibility of a direct role for Notch signaling in regulating the *slit-1*-expressing midline.

We also examined the expression of *miR-124* in both *notch-2^+^* and *slit-1^+^* cells. We found co-expression of *notch-2* and *miR-124c* in the periphery of the brain in uninjured animals (Fig. 7G,G′). During head regeneration, a subset of *miR-124^+^* cells colocalized with *slit-1* at 3 dpa, but not at 7 dpa (Fig. 7H,H′, Fig. S9K). These expression dynamics suggest that *miR-124* has the potential to function cell-autonomously in *notch-2^+^* and *slit-1^+^* cells.

Cumulatively, these phenotype and expression data suggest that the expansion of the *slit-1*-expressing cell population observed after *miR-124* KD may be mediated via direct regulation of *notch-2* and subsequent canonical Notch signaling in planarians. Moreover, this indicates that our list of predicted targets of *miR-124* promises to be extremely useful for further dissecting the complex roles of this miRNA family in the regeneration of the brain, visual system and midline of planarians.

## DISCUSSION

Although several genes crucial for anterior fate determination and nervous system regeneration have been identified (Vasquez-Doorman and Petersen, 2014; Fraguas et al., 2014; Blassberg et al., 2013; Roberts-Galbraith and Newmark, 2013; Agata and Umesono, 2008; Beane et al., 2011; Higuchi et al., 2008; Gurley et al., 2008; Rink et al., 2009; Bonar and Petersen, 2017; Seebeck et al., 2017; Currie et al., 2016; Wang et al., 2016; Roberts-Galbraith et al., 2016; Adell et al., 2010), how these genes are coordinated in time and space to establish the developmental transitions necessary for form and function of the nervous system remains to be fully elucidated. Our study has revealed enrichment for the *miR-124* family of miRNAs in the nervous system of planarians during homeostasis and discrete phases of head regeneration*.* The expression of *miR-124c* and *miR-124b* observed in anteriorly regenerating tissue at 3 dpa (Fig. 1E) suggests that these miRNAs might play a role in the organization of the brain and the patterning of visual neurons, rather than in specification of the brain primordium. We tested this hypothesis by perturbing the function of *miR-124* during planarian regeneration.

To perform loss-of-function studies for *miR-124* in planarians, we developed a novel vehicle to deliver anti-miRs using co-liposomes (Fig. 2). This method proved superior to soaking and injection, and we observed a comparably higher success rate for miRNA-mediated and also dsRNA-mediated KD using co-liposomes (Fig. 3, Fig. S5). *miR-124* KD animals regenerating anterior tissue had either reduced or absent photoreceptors. Interestingly, the posterior regenerating tissue and the intact animals did not show any regeneration defects. However, the lack of homeostatic defects in *anti-miR-124* KD animals might simply reflect slow turnover rates for neural cell types and/or the targeted mRNAs and proteins.

**Fig. 8.**
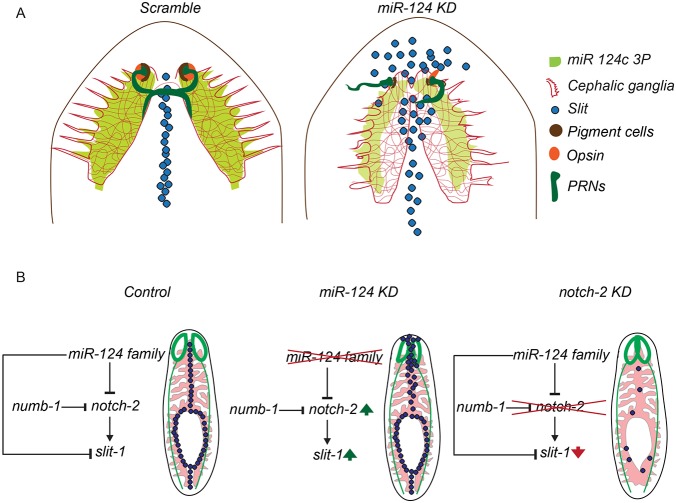
**Summary of *miR-124* function during planarian regeneration.** (A) Schematic of the brain, eye and midline after head regeneration in *miR-124* KD versus control planarians. KD of *miR-124* causes a reduction in the size of the cephalic ganglia, a loss of eye cells, disorganization of the optic chiasm, and an expansion of the *slit-1^+^* midline population. (B) Proposed genetic interaction in which *miR-124* modulates *slit-1* via inhibition of *notch-2* expression. The schematic also summarizes the organization of the regenerated brain (green), gut (pink) and *slit-1^+^* midline (black) following KD of *miR-124* or *notch-2*.

Extensive characterization of *miR-124* KD planarians using various brain- and eye-specific markers revealed diverse defects in the organization of the brain and visual neurons. The diverse phenotypes observed might be attributed to variability in the extent of KD or to changes in the large cohort of genes with varied functions in *miR-124* KD animals. Our study also revealed the key role of *miR-124* in regulating neural subtype specificity and organization during planarian regeneration. For instance, *miR-124* KD led to a reduction in the number of GABAergic and dopaminergic neurons, in addition to an increase in cholinergic neurons. However, the pathway through which *miR-124* regulates neural subtype specificity is not known. Nonetheless, our data provide robust evidence for an important role for *miR-124* in brain organization, the generation and/or maintenance of eye progenitors, and visual system patterning in regenerating planarians (Fig. 8A). Furthermore, our work also highlights the need to invest time and effort in developing novel liposome vehicles, which might help advance the development of transgenic methods in planarians.

Target prediction revealed a cohort of genes involved in PCP (*dishevelled-2*, *daam-1*), axon guidance (*netrin-1*, *slit-1*, *dscam*) and the Notch pathway (*notch-2*, *delta-3*, *serrate-1*) as predicted targets of *miR-124*. Interestingly, *miR-124* target conservation across the metazoans revealed that some of the planarian *miR-124* targets, particularly *notch-2*, *netrin-1* and *slit-1*, are also targets of *miR-124* in vertebrates, but not in flies and nematodes (Fig. S11, Table S4). This is a notable difference given the extensive functional conservation in neurogenesis ascribed to *miR-124* (Cao et al., 2006, 2007). There might be several biological and methodological reasons for this difference. First, it might be that only vertebrates have evolved the ability for *miR-124* to target *notch*, *netrin* and *slit* orthologs, and that the target predictions obtained for planarians might be an artifact. The evolutionary argument seems unlikely given that, in the invertebrate *Ciona intestinalis*, a feedback interaction between *miR-124* and Notch signaling regulates the epidermal-peripheral nervous system (PNS) fate choice in tail midline cells (Chen et al., 2011). These findings suggest that our target predictions are not only biologically relevant, but also that they point to an ancestral origin of the regulation of the Notch pathway by the *miR-124* family of miRNAs. Such a regulatory nexus might have been lost in *C. elegans* and *D. melanogaster*.

Among the putative *miR-124* targets, three genes encoding axon guidance proteins were uncovered. *netrin-1* and *dscam* are expressed in the planarian brain, while *slit-1* is expressed in the midline along the length of the A/P axis of planarians (Yamamoto and Agata, 2011; Fusaoka et al., 2006; Cebria et al., 2007). The spatial and temporal expression of these genes is essential for proper patterning of the brain and the visual neurons. For instance, perturbation of *slit-1* expression in the midline resulted in the collapse of the photoreceptors, demonstrating the key role for *slit-1* in proper patterning of the visual neurons to form the optic chiasm. Furthermore, our expression study based on double FISH for *slit-1* and *miR-124* in regenerating animals revealed spatial and temporal changes in the expression of these genes in the midline region of anteriorly regenerating tissue. Thus, we hypothesize from these data that *miR-124* expression in the brain might be essential for regulation of *slit-1* expression during anterior regeneration. This hypothesis was supported by the *miR-124* KD studies, which showed increased levels of *slit-1* expression in the anterior region of the regenerating planarian. However, the expression of *miR-124* is neuronal, except at 3 dpa when *miR-124* was seen in *slit-1^+^* cells, whereas the expression of *slit-1* is non-neuronal. This raises an important question regarding the molecular mechanism by which *miR-124* regulates the expression of *slit-1*. Our study has demonstrated that KD of *notch-2* and *delta-3* led to downregulation of *slit-1*, and KD of its negative regulator *numb-1* caused ectopic expression of *slit-1.* The *notch/numb* KD studies were supported by colocalization studies, which showed co-expression of *delta-3* and *notch-2* in *slit-1^+^* cells, suggesting that a NOTCH-2–DELTA-3 interaction might regulate *slit-1* expression. Similarly, *miR-124* KD led to increased levels of *notch-2* expression and ectopic expression of *slit-1* in the anterior region of the planarians (Fig. S8C, Fig. S6D). These results were further supported by colocalization studies using FISH, which revealed co-expression of *notch-2* and *miR-124* in the periphery of the brain. Together, these results suggest a role for *miR-124* in modulating *notch-2* expression, which is required for regulating *slit-1* in the midline of the anterior regenerating tissue. However, it is still not clear how *miR-124* expression in the brain regulates the *notch-2* domain crucial for *slit-1* expression. Recent studies have shown that miRNAs can be secreted from cells after packaging into exosomes, allowing delivery to adjacent cells and target suppression (Lee et al., 2017). The possibility of an exosome-mediated mechanism to deliver *miR-124* to the *slit-1^+^* cells needs to be investigated further. Nonetheless, based on our results, we propose a mechanism by which *miR-124* regulates *slit-1* expression via modulation of canonical Notch signaling (Fig. 8B).

The target prediction data from *miR-124* KD animals also revealed the PCP genes *dishevelled-2* and *daam-1* as potential targets of *miR-124*. In planarians, *vang-1*, *daam-1* and *ROCK* KD results in supernumerary eye formation and hyperplasia of neurons throughout the body (Beane et al., 2012). Our results suggest that *miR-124* KD animals behave like gain-of-function mutants of the PCP pathway, with premature termination of neural growth that leads to a significant decrease in the size of the cephalic ganglia and eyes. However, a rigorous target validation and dissection of the PCP pathway in planarians is necessary to establish whether the *miR-124* family plays a role in regulating this important developmental pathway.

In summary, we report on the development of a novel vehicle to deliver exogenous nucleic acids into planarian cells consisting of co-liposomes designed to have high fusogenic properties with planarian cell membranes. By combining these liposomes with anti-miRs, we were able to robustly target and knock down the function of *miR-124*, resulting in cephalic regeneration phenotypes. Our study reveals the key role of *miR-124* in neuronal organization and axon guidance during regeneration of the brain and visual system in planarians. Such a role for *miR-124* might be found in other neuronal regeneration paradigms.

## MATERIALS AND METHODS

### Animal maintenance

*Schmidtea mediterranea* planarians were maintained in 1× Montjuic solution at 20°C. Animals were starved for a week prior to the experiments, as described previously (Sasidharan et al., 2013).

### *In situ* hybridization and immunostaining

Whole-mount *in situ* hybridization (WISH) was performed as previously described (Pearson et al., 2009; Sasidharan et al., 2013; King and Newmark, 2013). miRNA-based WISH was carried out using digoxigenin-labeled miRCURY LNA probes obtained from Exiqon. For immunostaining, animals were fixed using Carnoy's solution as described (Sánchez Alvarado and Newmark, 1999). Rabbit anti-ARRESTIN (1:5000, clone VC-1; gift of Dr Kiyokazu Agata, Kyoto University, Japan) and mouse anti-SYNORF-1 (1:100, DHSB) primary antibodies were used. Species-specific secondary antibodies were obtained from Molecular Probes (1:400). Hoechst 33342 (25 µg/ml; Sigma) was used as a nuclear counterstain. Animals were mounted with Mowiol mounting medium (Sigma) and stored at 4°C until imaging.

### Preparation and biophysical characterization of liposomes

Liposomes were prepared with 1:1 molar ratios of lipid and cholesterol at a total concentration of 2 mM. Dry thin films of lipids were prepared in glass vials, followed by overnight hydration with deionized water to produce hydrated films. Vortexing vials for 2-3 min produced multilamellar vesicles. Subsequently, bath sonication (Qsonica sonicator, Q700) produced small unilamellar vesicles. The size and zeta potentials of liposomes (2 mM) were measured using a Zetasizer Nano ZS (Malvern). Using the same method, liposomes with planarian lipids and dual fluorescent lipids (1% of FRET pair, NBD-PE and N-Rho-PE) were made. Fluorescence intensities were recorded by excitation at 485 nm and emission at 530 nm. For the gel retardation assay, Cy3-labeled miRNA (2 μM) was complexed with liposomes in a total volume of 30 μl in HEPES buffer (pH 7.4). The samples were electrophoresed at 80 V for 20 min and the miRNA bands were visualized using an agarose gel documentation unit. Each of the methods used for the biophysical characterization of liposomes is described in more detail in the supplementary Materials and Methods.

### Knockdown of *miR-124*, *ovo* and Notch pathway genes

For *miR-124* and *ovo* KD, sexual planarians were pre-pharyngeally amputated, and the head and tail fragments were treated separately with liposome-complexed anti-miRs and scrambled miRs. Notch pathway gene KD was by injecting and feeding dsRNA to intact planarians followed by amputation. Detailed protocols are provided in the supplementary Materials and Methods.

### Transcriptome analysis

Total RNA was isolated from scrambled-treated and anti-miR-treated animals (*n*=15) using Trizol (Invitrogen) after two rounds of regeneration. The transcriptome library was prepared from the isolated total RNA using the TruSeq RNA Library Prep Kit v2 (Illumina) following the manufacturer's instructions. The transcriptome library was subject to paired-end sequencing (150 bp) on a NextSeq 500 next-generation sequencing machine (Illumina). We obtained 54.47 and 91 million reads from *miR-124c* KD and scrambled-treated samples, respectively. We used a reference-based transcriptome assembly algorithm TopHat v2.0.8, Cufflinks v1.0.3 pipeline to assemble the transcripts, with the Ox_Smed_v1 transcriptome as the reference annotation to guide RABT (reference annotation based transcript) assembly. Please refer to the supplementary Materials and Methods for a detailed protocol.

### Real-time PCR

Sequencing data were validated by real-time PCR on an ABI PRISM 7900HT. Total RNA used for next-generation sequencing was further used for qPCR validation. Primers are detailed in Table S5. A detailed protocol is provided in the supplementary Materials and Methods.

### miRNA target prediction and validation

We used miRanda v3.3a (Enright et al., 2003) to predict targets of *miR-124a*, *b* and *c.* The targets were filtered based on the complementarity score (score ≥140) and free energy (ΔG≤–14 kcal/mol). Target validation was performed in HEK293T cells transfected with pMIR-REPORT plasmid (Addgene) containing the 3′-UTR of the target and pRL-SV40 containing the Renilla luciferase gene (Promega) as a transfection control. miRNA mimics were obtained from Eurogentec and diluted as per manufacturer's instruction. A detailed protocol is provided in the supplementary Materials and Methods.

### LNA detection probes, *miR-124* inhibitors and qPCR primers

*anti-miR-124* was designed against 16 nucleotides at the 5′ end of *miR-124*. Short probes were used to facilitate better penetrance and substantially reduce cell toxicity, as recommended by the manufacturer. This anti-miR was predicted to KD all *miR-124* family members because these miRNAs share the same seed region and differ by only two or three nucleotides outside the seed region. Please refer to the supplementary Materials and Methods for a detailed list of detection and qPCR probes.
